# Community health worker team integration in Medicaid managed care: Insights from a national study

**DOI:** 10.3389/fpubh.2022.1042750

**Published:** 2023-01-13

**Authors:** Ashley Wennerstrom, Catherine G. Haywood, Denise O. Smith, Dakshu Jindal, Carl Rush, Geoffrey W. Wilkinson

**Affiliations:** ^1^Center for Healthcare Value and Equity, School of Medicine, LSU Health, New Orleans, LA, United States; ^2^Department of Behavioral and Community Health Sciences, School of Public Health, LSU Health, New Orleans, LA, United States; ^3^Louisiana Community Health Outreach Network, New Orleans, LA, United States; ^4^National Association of Community Health Workers, Boston, MA, United States; ^5^Center for Community Health Alignment, University of South Carolina, Columbia, SC, United States; ^6^Community Resources, LLC, San Antonio, TX, United States; ^7^Boston University School of Social Work, Boston, MA, United States

**Keywords:** community health workers (CHWs), Medicaid, managed care organization (MCO), integration, social determinants of health

## Abstract

**Introduction:**

Community health workers (CHWs) have historically worked in community-based settings. Medicaid managed care organizations (MCOs) are integrating CHWs into their teams, largely to support social determinants of health. Little is known about how teams are structured in these environments or how CHWs and their supervisors perceive CHW roles in MCOs.

**Methods:**

In 2021, two CHW professional associations and a university partnered to conduct a national cross-sectional survey of CHWs working with MCOs.

**Results:**

A total of 146 CHWs representing 29 states and 55 supervisors working in 34 states completed the survey. Although two-thirds of supervisors said only a high school diploma or equivalent was required for hiring, over half of CHWs reported having a bachelors or graduate degree. The majority of CHWs (72.6%) and employers (80%) said CHWs receive training in core competencies. Under half of CHWs reported working with a registered nurse (RN) (45.8%) or social worker (43.8%), and about a third work with a behavioral health (36.3%) or primary care provider (33.6%). Among supervisors, 70.9% identified social workers as CHWs' team members and over half indicated CHW work with RNs (56.4%), behavioral health (54.5%) and primary care providers (52.7%). Over half of CHWs (52.1%) and roughly two thirds (63.6%) of supervisors indicated that CHWs use electronic health records. Roughly 85% of CHWs make referrals and roughly three quarters conduct social screenings. Around half of CHWs said they assist with care planning (54.1%), conduct health screenings (52.1%) or participate in case reviews (49.3%). About three quarters of CHWs (75.3%) and over two thirds of supervisors (67.3%) believed that CHWs are utilized to their full potential. Under three quarters of CHWs (72.6%) and over half of supervisors (54.4%) believe CHWs are equitably compensated for their work.

**Discussion:**

Overall, CHWs roles in MCOs appear to focus on supporting clinical care and making referrals for social issues, rather than addressing community-level concerns. Health plans should ensure that CHWs have the professional freedom to develop community-based solutions to common social needs. MCOs should also ensure that CHWs receive equitable compensation and ensure that CHWs have opportunities for promotion.

## Introduction

Community health workers (CHWs) are a critical frontline public health workforce defined by their trusted relationships with the communities they serve (1). Operating under a number of job titles including *promotores de salud* and community health representatives, for at least 60 years, they have linked individuals and communities with health and social services, helped people navigate complex systems, and advocated for structural changes in policies related to social determinants of health (2–4). While CHWs' roots are primarily in social justice focused, community-based settings, their work has expanded to a variety of agencies including federally qualified health centers, health departments, and universities, among others (5). There is a nationally recognized set of CHW roles and competencies (6), but programs employing CHWs vary based on community strengths and needs, as well as employer structure, focus, capacity, and funding limitations. Although CHWs are difficult to enumerate, in part due to their various job titles, as of 2021, an estimated 61,000 CHWs worked throughout the United States (7).

In the last decade, as health systems have begun to understand the necessity of addressing social issues, many have moved to integrate CHWs into their care teams. The addition of CHWs to clinical service delivery has been found to improve health outcomes (8) and enhance the patient experience of care (9), and support access to culturally appropriate care (10). For example, integrating CHWs into patient-centered medical homes helps teams better understand patients' backgrounds, challenges, and preferences related to care, which can help improve health outcomes (11). Hospital readmission rates have been reduced among patients who receive care from teams with CHWs (9, 12). An investigation of over a hundred innovative care delivery models revealed that only those using CHWs lowered cost (13). There is also some evidence that adding CHWs to clinical care teams has improved adherence to medications and timely utilization of healthcare services (14).

Researchers seeking to understand factors that promote successful integration have identified clarity of team member roles, clearly defined workflows, and positive culture as important (14, 15). Healthcare team members, including CHWs, have reported that the presence of leaders who support CHWs, as well as a clinic culture that focuses on social, rather than exclusively medical needs, is also critical (16). However, there is some evidence that integration can present challenges to CHWs in maintaining their unique identities as community advocates (17). Healthcare settings tend to value formal education and training above lived experience when hiring CHWs (17), raising questions about whether clinical integration may pull CHWs away from their roots in social justice.

Another area in which CHWs newly find themselves engaged is in Medicaid managed care. Managed care organizations (MCOs), which enter contracts with states to provide health services to Medicaid members, usually on a per member per month basis (18, 19), are highly motivated to achieve two aims in which CHWs are skilled: improving outcomes and reducing costs of care (13, 20–23). MCOs generally have flexibility in their staffing and service delivery models, and some have opted to hire CHWs or contract with external organizations for CHW services. With an increasing emphasis on addressing population health, some states have started to require that MCOs employ CHWs. As of 2021, 10 of 41 states (including DC) that have managed care have instituted some sort of requirement that their contracted MCOs offer CHW services to enrollees. An additional six states indicated that they would also require CHW services in their contracts in the following year (24). As one example, managed care contracts in New Mexico require that at least 3% of enrollees must receive CHW support (25).

Although there is clear momentum for integrating CHWs into MCOs, there is a dearth of national information about MCO priorities for hiring and training CHWs, how teams are structured in these environments, and how CHWs and their supervisors perceive CHW participation in work with MCOs. A few state-level studies provide some important insight. New Mexico-based Molina Healthcare, an early adopter of CHWs employment in an MCO, provided a week of training in many core CHW skills and hired CHWs to support frequent users of the emergency department through education, social support and advocacy, resulting in reduced emergency department visits and overall costs of care (22). In California, providers reported positive experiences when collaborating with CHWs through an MCO initiative that focused on ensuring that CHWs hired held credibility in their communities (26). A 2018 study found that hiring practices and qualifications for CHW employment varied widely among MCOs in Arizona (27) and coordination care organizations in Oregon have found that a lack of understanding of CHW roles among leadership has proved a barrier to CHW integration (28).

This national study aimed to add to the evidence base regarding CHW-MCO integration by surveying CHWs about their experiences working with MCOs. CHW program supervisors working with MCOs were also surveyed, as supervision is critical to successful CHW-team integration (29). Survey questions focused on employer-offered training, CHW responsibilities within their teams, team structure and supervision, reporting structure, and perceptions of team integration. The research team expected that CHWs roles and responsibilities within MCOs would largely be focused on improving clinical outcomes.

## Materials and methods

A researcher from LSU Health Sciences Center—New Orleans and CHWs from the Louisiana Community Health Outreach Network (LACHON) and the National Association of Community Health Workers (NACHW) with longstanding relationships collaborated to carry out this study. Two subject matter experts who are founding board members of NACHW provided additional guidance.

The research team based its study methods on previous recommendations for conducting CHW workforce survey research, which include engaging CHWs in survey design, collaborating with CHW networks to distribute the survey, and piloting the survey with CHWs (30). The team collaboratively agreed upon a general list of survey topics including demographics, CHW responsibilities, team structure, and perceptions of team integration. Working from a prior survey (31), the team then selected relevant questions to address the topics identified. Questions and response categories were updated and added, as needed.

The survey was distributed online *via* LACHON's listserv of over 400 CHWs and allies and through NACHW's member newsletter. It remained open from March to July 2021. Over 20 local, state, and regional CHW networks and associations, as well as a dozen national organizations (e.g., policy-focused think tanks and trade organizations for health insurers), were enlisted to support survey distribution. Criteria for participation included: (1) being an adult (18+ years of age) and (2) being employed as a CHW or CHW supervisor at an MCO or at another organization (e.g., a community-based organization) that receives a contract from an MCO to provide CHW services. Interested participants were entered into a raffle for a pre-paid $50 Visa gift card.

Informed consent language was included at the beginning of the survey. The IRB at LSU Health Sciences Center reviewed and approved all research procedures. All data were analyzed using SPSS version 26. Descriptive statistics are reported.

## Results

A total of 146 CHWs representing 29 states and 55 supervisors working in 34 states (among 41 with managed care) completed the survey. Among CHWs, over one quarter (27.4%) of respondents were from the West. Just under one quarter (22.6%) reported being from the Midwest. Another 18.5% worked in the Mid-Atlantic region, while 16.4% were from the South, and 15.1% the Northeast. Supervisors often worked across state lines, with 45.5% working in the South and over four in 10 (41.8%) working in the Midwest. Almost one third (30.9%) had work activities in the West, while another 14.5% were in the Northeast. Just 10.9% reported working in the Mid-Atlantic region.

The vast majority of CHWs (87%) and supervisors (76.4%) were women. About four in 10 CHWs were Black, 30.8% were white, and roughly one-quarter were Hispanic/Latinx. Almost six in 10 supervisors were white, just under a quarter were Black, and 7.3% were Hispanic/Latinx. CHWs in this sample most commonly reported having completed some college (37.7%) or a bachelor's degree (37.7%). Nearly three-quarters of the supervisors had a graduate degree. Demographics are summarized in Table 1.

**Table 1:** Demographics of national sample of CHWs and CHW supervisors working with Medicaid managed care organizations.

**Variable**	**CHW**	**Supervisor**
	**(*N =* 146)**	**(*N =* 55)**
Age, range	22–72	24–70
Age, mean (SD)	43.8 (12.6)	45.32 (11.9)
	***n*** **(%)**	***n*** **(%)**
**Gender**		
Woman	127 (87.0)	42 (76.4)
Man	12 (8.2)	10 (18.2)
Prefer not to identify	2 (1.4)	1 (1.8)
No response	5 (3.4)	2 (3.6)
**Race/ethnicity**		
African American/Black	58 (39.7)	13 (23.6)
White	45 (30.8)	32 (58.2)
Hispanic/Latinx	37 (25.3)	4 (7.3)
Native American/AI	6 (4.1)	0 (0.0)
Asian	2 (1.4)	4 (7.3)
Native Hawaiian/Pacific Islander	1 (0.7)	0 (0.0)
Another race	1 (0.7)	1 (1.8)
**Education**		
Less than high school	2 (1.4)	0 (0.0)
High school or GED	9 (6.2)	0 (0.0)
Some college or 2-year degree	55 (37.7)	5 (9.1)
Bachelor's degree	55 (37.7)	7 (12.7)
Graduate or professional degree	20 (13.7)	41 (74.5)
No response	5 (3.4)	2 (3.6)

In response to a question about professional requirements for hiring CHWs, just under 10% of supervisors said there was no minimum. About two-thirds of supervisors said a high school diploma or equivalent was necessary. Roughly 9% looked for some college or an associate's degree and about 11% required a bachelor's degree. Table 2 contains these results.

**Table 2:** Minimum CHW education required for hiring as reported by supervisors working with Medicaid managed care organizations.

**Education**	**Supervisor (*N =* 55)**
	***n* (%)**
No minimum	5 (9.1)
High school or equivalent	37 (67.3)
Some college	3 (5.5)
Associates	2 (3.6)
Bachelors	6 (10.9)
No response	2 (3.6)

The vast majority of CHWs (72.6%) and employers (80%) reported that CHWs receive training in core competencies. Over half of CHWs indicated that they received training in motivational interviewing and advocacy from their employer. Results are detailed in Table 3.

**Table 3:** Employer-offered training for CHWs reported by a national sample of CHWs and CHW supervisors working with Medicaid managed care organizations.

**Topic**	**CHW**	**Supervisor**
	**(*N =* 146)**	**(*N =* 55)**
	***n* (%)**	***n* (%)**
Core competencies	106 (72.6)	44 (80.0)
Motivational interviewing	86 (58.9)	46 (83.6)
Advocacy	73 (50.0)	35 (63.6)
Specific health topic	69 (47.3)	40 (72.7)
Chronic disease	64 (43.8)	35 (63.6)
Navigation	56 (38.4)	29 (52.7)
Peer support	48 (32.9)	26 (47.3)
Leadership	47 (32.2)	22 (40.0)
Medical interpretation	24 (16.4)	8 (14.5)
Languages	10 (6.8)	7 (12.7)

In terms of team structure, around 8 in 10 CHWs (82.2%) and supervisors (78.2%) indicated that CHWs collaborate with other CHWs. Over half of CHWs and nearly three quarters of supervisors said there was a program manager or director involved. Nearly half of CHWs (47.0%) and about 6 in 10 supervisors (58.2%) said case managers were part of teams. With regard to clinical staff, just under half of CHWs indicated working with a registered nurse (RN) (45.8%) or social worker (43.8%) and about a third said they work with a behavioral health (36.3%) or primary care provider (33.6%). Supervisors more frequently endorsed clinical staff as members of teams, with 70.9% identifying social workers as team members and over half indicating RNs (56.4%), behavioral health (54.5%) and primary care providers (52.7%). Around one in six in both groups indicated that CHWs collaborate with dieticians or nutritionists.

CHWs and employers largely agree that supervision is most commonly provided by a program manager. Fully one quarter of supervisors indicated that social workers supervise CHWs, in contrast to just 5% of CHWs. About one in six CHWs listed another job title as a supervisor. These included the clinic manger, manager of population health, and chief operating officer.

In terms of work documentation, 72.6% of CHWs and 63.6% of employers indicated that CHWs meet with supervisors. About two thirds of CHWs (63.0%) and half (49.1%) of supervisors reported that CHWs use an internal database to track activities. Just over half of CHWs (52.1%) and roughly two thirds (63.6%)of supervisors said that CHWs use electronic health records. About a third of both groups noted that CHWs use narrative reports. These results are summarized in Table 4.

**Table 4:** Team structure and reporting methods among a national sample of CHWs and CHW supervisors working with Medicaid managed care organizations.

**Variable**	**CHW ** **(*N =* 146)** ***n* (%)**	**Supervisor** **(*N* = 55)** ***n* (%)**
**Team members**		
CHWs	120 (82.2)	43 (78.2)
Program manager/director	83 (56.8)	40 (72.7)
Case manager	70 (47.9)	32 (58.2)
RN	66 (45.8)	31 (56.4)
Social worker	64 (43.8)	39 (70.9)
Behavioral health provider	53 (36.3)	30 (54.5)
Primary care provider	49 (33.6)	29 (52.7)
Dietician/Nutritionist	24 (16.4)	10 (18.2)
Other	10 (6.8)	5 (9.1)
**CHW supervisor**		
Program manager	80 (55.9)	38 (69.1)
Team leader/director	17 (11.6)	12 (21.8)
Senior CHW	11 (7.7)	8 (14.5)
Social worker	7 (4.9)	14 (25.5)
Case manager	4 (2.8)	8 (14.5)
Other	24 (16.4)	7 (12.7)
**Methods of reporting CHW activities**		
Supervisor meetings	106 (72.6)	35 (63.6)
Database	92 (63.0)	27 (49.1)
Electronic health record	76 (52.1)	35 (63.6)
Narrative reports	51 (34.9)	18 (32.7)
Other	8 (5.5)	2 (3.6)

Making referrals was the most common responsibility that CHWs indicated. About three quarters of CHWs indicated that they receive referrals and conduct social screenings while 85.5% of supervisors endorsed such activities. Over 6 in 10 CHWs and 7 in 10 supervisors said that CHWs conduct home visits. Around half of CHWs said they assist with care planning (54.1%), conduct health screenings (52.1%) or participate in case reviews (49.3%). Supervisors' reports of these activities were all slightly higher. CHWs and employers alike reported that < 3 in 10 CHWs provide medical interpretation. Results are reported in Table 5.

**Table 5:** CHW responsibilities reported by a national sample of CHWs and CHW supervisors working with Medicaid managed care organizations.

**CHW responsibilities**	**CHW** **(*N =* 146)** ***n* (%)**	**Supervisor** **(*N =* 55)** ***n* (%)**
Make referrals	124 (84.9)	40 (81.8)
Receive referrals for education or other support	111 (76.0)	47 (85.5)
Conduct social screenings	110 (75.3)	47 (85.5)
Receive referrals for home visits	89 (61.0)	39 (70.9)
Assist in developing or coordinating care plans	79 (54.1)	34 (61.8)
Conduct health screenings	76 (52.1)	31 (56.4)
Participate in case reviews	72 (49.3)	35 (63.6)
Provide medical interpreting services	41 (28.1)	16 (29.1)
Other	9 (6.2)	2 (3.6)

Roughly 93 percent of CHWs and supervisors agreed that CHW work is valued at their organization. Nine in 10 CHWs and 94.5% of supervisors agreed that supervisors understand the work CHWs do. Over eight in 10 CHWs and supervisors indicated that CHWs are well-integrated into teams. Almost nine in 10 CHWs believed their teams understand their work (89.0%) and that they are valued (85.6%). Similarly, 83.6% of supervisors agree that CHWs roles are understood and 89.1% believe CHWs are valued by other team members. About three quarters of CHWs (75.3%) and just over two thirds of supervisors (67.3%) believed that CHWs are utilized to their full potential. Just under three quarters of CHWs (72.6%) and over half of supervisors (54.4%) indicated that CHWs are equitably compensated for their work. Among both CHWs and supervisors, about 6 in 10 agreed that CHWs have opportunities for promotion. These results are detailed in Table 6.

**Table 6:** Perception of CHW value and team integration among a national sample of CHWs and CHW supervisors working with Medicaid managed care organizations.

**CHW responses** ***N** **=*** **146**				**Supervisor responses** ***N** **=*** **55**			
**Statement**	**Agree completely/ somewhat** ***n*** **(%)**	**Disagree completely/ somewhat** ***n*** **(%)**	**N/a** ***n*** **(%)**	**Statement**	**Agree completely/ somewhat** ***n*** **(%)**	**Disagree completely/ somewhat** ***n*** **(%)**	**N/a** ***n*** **(%)**
My organization values the work that I do	135 (92.5)	7 (4.8)	0 (0.0)	My organization values the work CHWs do	51 (92.7)	2 (3.6)	0 (0.0)
My supervisor understands the work that I do	131 (89.7)	9 (6.2)	1 (0.7)	CHWs' work and roles are understood by the individuals who supervise them	52 (94.5)	0 (0.0)	0 (0.0)
The team I work with understands the work I do	130 (89.0)	12 (8.2)	0 (0.0)	CHWs' work and roles are understood by the teams they work with	46 (83.6)	7 (12.7)	0 (0.0)
I am a valued member of the teams I work with	125 (85.6)	14 (9.6)	2 (1.4)	CHWs are valued members of the teams they work with	49 (89.1)	4 (7.3)	0 (0.0)
I am well-integrated into the team at my organization	122 (83.6)	18 (12.3)	1 (0.7)	CHWs are well-integrated into team	45 (81.8)	7 (12.7)	0 (0.0)
I am utilized to my full potential	110 (75.3)	31 (21.2)	1 (0.7)	CHWs are utilized to their full potential	37 (67.3)	15 (27.3)	1 (1.8)
I am equitably compensated for my work	106 (72.6)	34 (23.8)	1 (0.7)	CHWs are equitably compensated	30 (54.5)	20 (36.4)	3 (5.5)
I have opportunities for promotion at my organization	87 (59.6)	44 (30.1)	11 (7.5)	CHWs have opportunities for promotion	35 (63.6)	14 (25.5)	4 (7.3)

Totals may not sum to 100% due to missing responses.

## Discussion

This study examined the responsibilities, team structure, and perceptions of integration among MCO-supported CHWs and their employers.

The sample of CHW respondents is similar to other studies of CHWs working across sectors, in that the majority are women and people of color (32). In terms of hiring CHWs, MCOs largely report they are not imposing formal educational requirements beyond high school, but the educational level reported by CHWs—with just 7.6% having a high school education or less and over half having a college or graduate degree—suggests that in practice, MCOs prioritize hiring people with higher levels of education. This finding is concerning, given that CHWs' primary qualification has always been community trust. It also suggests that those making hiring decisions may be unfamiliar with CHWs, which is consistent with a prior study (28).

Overall, there are several indicators that CHWs roles are being conceptualized in terms of supporting clinical care. Supervisor responses to the types of training CHWs receive, which largely included CHW training in skills such as motivational interviewing, chronic disease, and navigation, are directly related to helping patients manage chronic conditions and, ultimately, reducing costs of care. In addition, CHWs' use of electronic health records, along with their participation in conducting case reviews and developing care plans alongside clinically trained providers demonstrate that CHWs are largely focused on improving health outcomes among individual Medicaid members. This medicalized approach suggests that CHWs may have limited time to engage in more community-based, social justice work that is the historical hallmark of the profession (2, 4), and appears to be consistent with a prior study of coordinated care organizations that found individually-focused CHW activities were more common than community-level advocacy (28).

The finding that CHWs make and receive referrals is consistent with nationally recognized CHW roles (6). Although a study in 2018 found limited CHW engagement in conducing assessments (27), the finding that CHWs are now conducting screenings for social needs is unsurprising, given the increasing interest in addressing social determinants of health and that some state Medicaid contracts specifically outline that role (24). As MCOs continue to engage CHWs in their service delivery models, it will be critical to ensure that CHWs' efforts to address social determinants of health are not limited to merely making referrals for social issues identified through screening. CHWs will need freedom to not only develop relationships with agencies that receive member referrals, but also to develop community-based solutions to common social needs. For example, CHWs may collaborate with one another and local leaders to develop a food bank in an area with limited resources. CHWs working in teams have identified the opportunity to network as being critical to their roles (33). Furthermore, ensuring that CHWs have the flexibility to respond to community-level issues could help address the substantial proportion of CHWs and supervisors who report that CHWs are not utilized to their full potential.

It is encouraging that CHWs and supervisors alike generally perceive that CHWs roles are understood and valued by fellow team members. However, it is concerning that over one quarter of CHWs do not feel that CHWs are equitably compensated, as do nearly one half of supervisors, who likely have greater insight than CHWs into compensation levels for various positions. Broadly, CHW contributions have often been conceptualized in terms of return on investment (i.e., how does the cost of CHW salaries, benefits, and supervision compare to costs saved through reduced health care services use?), and MCOs may be developing CHW salary scales based on this approach. If so, they would be wise to consider that much of the value CHWs bring in terms of addressing health related service needs issues (e.g., education, housing, food, transportation, re-entry) for individuals and families may not immediately be reflected in healthcare costs. As health financing reform shifts risk to providers and drives care “upstream,” equitable compensation for CHWs, reflecting their value in addressing social determinants of health and promoting health equity, should be considered a prudent investment. Fair compensation may also be an important step toward addressing the inherent power differential between CHWs and clinically trained providers.

In addition to concerns about salaries, health plans should make efforts to address the substantial proportion of CHWs and supervisors who do not perceive that CHWs have opportunities for promotion. It is worth noting that CHWs are largely being supervised by people who are not CHWs, and likely do not have experience in the field. MCOs might consider collaborating with CHWs to develop career pathways (e.g., promotion to CHW supervision or program management) to ensure that CHWs do not perceive themselves to be in “dead end” jobs.

## Limitations

This study has several limitations. Because it is a cross sectional study, it is not possible to draw causal inferences. The sample size is also relatively small. Due to the survey distribution method (i.e., requesting that CHW networks share the survey with their members), it is not possible to calculate a response rate because we cannot ascertain the number of CHWs who received the survey and met the inclusion criteria. There may also be some selection bias if the group that was most likely to receive the survey—those who are members of a professional network—has different characteristics than those unaffiliated with CHW networks. Statistical testing to assess differences between supervisors and CHWs was not conducted because members of each group did necessarily work at the same organizations (i.e., the supervisors who responded may not have supervised the CHWs who did). Furthermore, CHWs and supervisors are substantially different and would reasonably be expected to have different perspectives. Despite these limitations, the sample was nationally representative, and it sheds light on a topic that is under-studied.

## Conclusion

Overall, CHWs roles in MCOs appear to focus on supporting clinical care and making referrals for social issues, rather than addressing community-level concerns. Health plans should ensure that CHWs have the professional freedom to develop community-based solutions to common social needs. MCOs should also ensure that CHWs receive equitable compensation and ensure that CHWs have opportunities for promotion.

## Data availability statement

The datasets presented in this article are not readily available to protect the confidentiality of participants. Requests to access the datasets should be directed to awenne@lsuhsc.edu.

## Ethics statement

The studies involving human participants were reviewed and approved by LSU Health Sciences Center—New Orleans IRB. Written informed consent for participation was not required for this study in accordance with the national legislation and the institutional requirements.

## Author contributions

AW: conceptualization, methodology, investigation, formal analysis, writing—original draft, and funding acquisition. CH and GW: conceptualization, methodology, and data curation. DS: conceptualization, methodology, data curation, and writing—review and editing. DJ: data curation, writing—original draft, and writing—review and editing. CR: conceptualization, data curation, and writing—review and editing. All authors contributed to the article and approved the submitted version.
